# Persistence of Positive RT-PCR Results for Over 70 Days in Two Travelers with COVID-19

**DOI:** 10.1017/dmp.2020.450

**Published:** 2020-11-19

**Authors:** Theo-Ben Kandetu, Eric J. Dziuban, Kaveto Sikuvi, Rachel S. Beard, Reginald Nghihepa, Gerhard van Rooyen, Andreas Shiningavamwe, Ismael Katjitae

**Affiliations:** 1 Ministry of Health and Social Services, Windhoek, Namibia; 2 US Centers for Disease Control and Prevention, Windhoek, Namibia; 3 Namibia Institute of Pathology, Windhoek, Namibia

**Keywords:** coronavirus, COVID-19, infection control, patient isolation

## Abstract

The relation of continuing to test positive for severe acute respiratory syndrome coronavirus 2 (SARS-CoV-2) by reverse transcription real-time polymerase chain reaction (RT-PCR) to infectivity remains unclear, with numerous consequences. This report describes 2 patients with persistent viral detection by RT-PCR for 77 and 72 days, respectively, longer than other reported case-patients who were otherwise healthy.

The significance of continuing to test positive for severe acute respiratory syndrome coronavirus 2 (SARS-CoV-2) by reverse transcription real-time polymerase chain reaction (RT-PCR) and its relation to infectivity for SARS-CoV-2 remain unclear. Some studies have linked symptomatic disease, male sex, age greater than 60 years, and severe forms of the disease to longer periods of viral RNA detection by RT-PCR, whereas others have not replicated these findings.^1-3^ The average time from first positive to first negative RT-PCR results has been described as less than 4 weeks, although the actual infectious period is postulated to be shorter.^1-5^ Some reports describe individuals with viral RNA detection up to 63 days, or longer in rare cases of immunocompromised individuals, undefined clinical course, or recrudescence.^6-12^

Namibia’s first reported cases of the 2019 coronavirus disease (COVID-19) were announced on March 14, 2020. A married couple, a 34-year-old man (Patient 1) and a 24-year-old woman (Patient 2), both living in Spain, had traveled to Namibia by air. Patient 1 developed symptoms of fever, dry cough, and fatigue while in transit on March 10 and sought medical evaluation within hours of arriving in Namibia. Patient 2 had no symptoms. Both were tested by nasopharyngeal (NP) and oropharyngeal (OP) swabs on March 11 and were positive for SARS-CoV-2 by RT-PCR. After an initial retesting by both NP and OP swabs, all subsequent tests were by NP swab only. Neither person had preexisting medical conditions to increase risk for severe illness from COVID-19.

Both patients were kept in an isolation facility for patients with mild cases of COVID-19. The patients had contact only with health care providers in appropriate personal protective equipment. Patent 1’s symptoms fully resolved in 10 days after onset, and Patient 2 did not develop any symptoms. However, RT-PCR testing continued to show presence of viral RNA after more than 2 months of testing (Table 1). Based on availability, 2 assays were used in Namibia over the course of testing done in the country (Table 1): (1) the ModularDx Kit SARS-CoV (COVID-19) E-gene plus EAV control (TIB) (TIB MOLBIOL, Berlin, Germany, https://www.tib-molbiol.com/); and (2) the Detection Kit for 2019 Novel Coronavirus (2019-nCoV) RNA (PCR-Fluorescence Probing) (DA AN) (DA AN Gene Co. Ltd., Sun Yat-Sen University, Guangzhou, China, http://en.daangene.com/). Both of these assays have been independently evaluated on the Roche 480 LightCycler PCR platform using 50 positives and 100 negatives, and the sensitivity and specificity were found to be as follows: TIB gene target E sensitivity 100% (95% CI: 93, 100) and specificity 100% (95% CI: 96, 100), DA AN gene target ORF1 sensitivity 100% (95% CI: 93, 100) and specificity 96% (95% CI: 90, 98), and DA AN gene target N sensitivity 100% (95% CI: 93, 100) and specificity 98% (95% CI: 93, 99) (https://www.finddx.org/covid-19/sarscov2-eval-molecular/molecular-eval-results/). All testing included internal controls, as well as extraction, positive, and negative controls to ensure testing quality and valid results. Cycle threshold values were not consistently reported, and a viral culture was not able to be performed.

**Table 1.** Testing collection dates, assay information, and testing outcomes of Patients 1 and 2

Date Tested	RT-PCR Assay Used	Patient 1 Result	Patient 2 Result
11-Mar-20	Unknown^a^	Detected	Detected
18-Mar-20	TIB	Detected	Detected
29-Mar-20	TIB	Detected	Detected
7-Apr-20	TIB	Detected	Detected
15-Apr-20	TIB	Not detected	Detected
17-Apr-20	TIB	Detected	Detected
22-Apr-20	TIB	Detected	Detected
24-Apr-20	DA AN	Detected	Not performed
27-Apr-20	DA AN	Detected	Not detected
27-Apr-20	DA AN	Detected	Detected
27-Apr-20	DA AN	Not detected	Not detected
4-May-20	DA AN	Detected	Detected
11-May-20	DA AN	Detected	Detected
16-May-20	DA AN	Not detected	Detected
19-May-20	DA AN	Not detected	Not detected
21-May-20	DA AN	Detected^b^	Detected
25-May-20	DA AN	Detected	Not performed
28-May-20	DA AN	Not detected	Not detected
30-May-20	DA AN	Not detected	Not detected

*Notes*: RT-PCR = reverse transcription real-time polymerase chain reaction; TIB = ModularDx Kit SARS-CoV (COVID-19) E-gene plus EAV control; DA AN = Detection Kit for 2019 Novel Coronavirus (2019-nCoV) RNA (PCR-Fluorescence Probing).

a First test was conducted in South Africa.

b Namibian protocol was to clear patients after 2 consecutive negative tests; however, Patient 1 had a new onset of sore throat and was re-tested.

Patient 1 had viral RNA detection by RT-PCR from the day after symptom onset (March 11) until 77 days after symptom onset (May 25). Patient 2 never had symptoms but had viral RNA detection by RT-PCR from March 11 to May 21 (72 days). Without clear reason, both patients exhibited detection of viral RNA for longer periods than previously reported in immunocompetent individuals, especially asymptomatic healthy persons such as Patient 2. Because of the possibility of cross-contamination, the couple was separated from each other in supervised isolation beginning on April 20, which was 38 days after their first positive test results. Among Namibia’s other patients diagnosed in March and April 2020, none had such extended periods of viral detection, and half (7/14) demonstrated negative RT-PCR results within 21 days of the initial positive result.

Additional published data regarding time to viral clearance and detection by RT-PCR will be valuable for public health officials and clinicians in their decision-making regarding patient isolation. More data are needed on the correlation between repeatedly testing positive for SARS-CoV-2 by RT-PCR and infectivity, viral load as a possible determinant, and the role of antigen tests or other accessible measures that can determine the presence of a viable virus.^13,14^ In settings where re-introduction of transmission to a community is of great concern, but isolation facility space is limited, a better understanding of infectivity duration would inform policies for use of limited resources and for reducing unnecessary burden on those infected.
